# Frequency of adenovirus serotype 8 in patients with Keratoconjunctivitis, in Ahvaz, Iran

**Published:** 2019-04

**Authors:** Kolsoom Shafiei, Manoochehr Makvandi, Ali Teimoori, Alireza Samarbafzadeh, Gholamreza Khataminia, Shahram Jalilian, Niloofar Neisi, Kimia Makvandi, Mehrdad Sadeghi Haj

**Affiliations:** 1 Infectious and Tropical Diseases Research Center, Health Research Institute, Ahvaz Jundishapur University of Medical Sciences, Ahvaz, Iran; 2 Department of Virology, School of Medicine, Ahvaz Jundishapur University of Medical Sciences, Ahvaz, Iran; 3 Department of Ophthalmology, Imam Khomaini Hospital, Ahvaz Jundishapur University of Medical Sciences, Ahvaz, Iran

**Keywords:** Human adenovirus, Polymerase chain reaction, Keratoconjunctivitis, Serotype

## Abstract

**Background and Objectives:**

Adenoviral keratoconjunctivitis is an extremely frequent ophthalmological disease caused by various serological subtypes of human adenovirus (HAdV) worldwide. Adenoviruses serotypes 8, 11, 19, 37 frequently cause epidemic keratoconjunctivitis (EKC). This study was conducted to evaluate the frequency of adenovirus serotypes in patients with EKC in Ahvaz, Iran.

**Materials and Methods:**

Eighty-eight ocular swabs were collected from patients with EKC. The specimens were analyzed for detection of adenovirus by standard PCR. The PCR products were further sequenced and analyzed to determine the serotypes.

**Results:**

The study population consisted of 49/88 (55.7%) males and 39/88 (44.3%) females. Among them 25 (51.02%) males and 22 (56.41%) females were positive for HAdV serotype 8 (*p*= 0.488). Overall forty-seven (53.4%) samples were positive for AdV serotype 8 while forty-one patients (46.59%) were negative for the adenovirus serotypes.

**Conclusion:**

The results of this study revealed predominanance of HAdV 8 with high prevalence of 53.4% among patients with Keratoconjunctivitis. Forty-one patients (46.59%) were negative for adenovirus. Still, the role for other related viruses such as enteroviruses need to be investigated in patients with EKC.

## INTRODUCTION

Epidemic keratoconjunctivitis (EKC) is a serious public health problem, caused by certain adenoviruses serotypes which have been reported in the different regions of the world (1, 2). EKC is a self-limited infection, the incubation period varies from 4 to 24 days. EKC is characterized by the involvement of the entire surface eye, including both the conjunctiva and corneal epithelia and hazy vision (3). Eye irritation, red eye, foreign body sensation, watery discharge and photophobia are the most common symptoms. The ocular signs are predominantly bulbar conjunctiva redness, blepharedema, epiphora, chemosis, follicular reaction, subconjunctival hemorrhage and membrane or pseudomembrane formation. It may lead to unilateral or bilateral follicular conjunctivitis followed by corneal subepithelial infiltrate, blurry vision and is associated with significant morbidity (4). EKC is highly contagious, and out-breaks have been reported in schools, military bases, and hospital wards. The modes of transmission are mainly through hand to eye contact, ocular secretions, respiratory droplets, and contact with ophthalmic care providers and medical instruments (1–3).

Human adenoviruses (HADVs) are non-envelope, icosahedral, double-stranded DNA viruses and are classified within the family *Adenoviridae*, genus *Mastadenovirus*, and are further divided into seven species (A–G) (5, 6).

Adenoviruses can cause an array of clinical diseases, including conjunctivitis, respiratory disease, hemorrhagic cystitis and gastroenteritis (7).

EKC and pharyngoconjunctival fever are the most common adenoviral ocular diseases (4, 8). Adenoviral keratoconjunctivitis is frequent ophthalmological disease and account for 15% to 70% of all conjunctivitis cases worldwide (3, 9).

Several adenoviruses including HAdV-8, HAdV-19 and HADV-37 (in HAdV-D) are responsible from severe and contagious EKC which can be related to a nosocomial infection or community acquired (4, 10–12).

Laboratory confirmation of the diagnosis can be useful tool for physicians in rapidly initiating suitable hygienic measures and determining the epidemiological significance of the infection. Diagnosis is clinically based on clinical features, and history symptoms. However, other causes of conjunctivitis such as chlamydial infection and herpes simplex virus or varicella zoster virus need to be excluded (9).

The hypervariable of the hexon region of HAdV is used for determination of adenovirus serotyping (4, 13, 14).

Detection of adenoviral DNA in conjunctival specimen is carried out by PCR which is reliable assay and commonly used in many laboratories across the world (4).

Adenovirus serotyping can be performed by sequencing or Restriction Fragment Lenght Polymorphism (RFLP) analysis. Identification of the adenovirus serotype involved in EKC is required to understand the geographical distribution of the adenovirus and to perfect the knowledge on the relation between a specific genotype and clinical features (4, 13). There is limited data regarding adenoviral kerato-conjunctivitis in Iran (9). The prevalence of EKC have been reported in different regions of the world (4, 13).

This study was aimed to detect and determine the adenovirus serotypes among the patients with EKC referred to Department of Ophtalmology, Imam Khomeini Hospital of Ahvaz city. Ahvaz city is the capital of Khuzestan province, located in the south-west region of the Iran.

## MATERIALS AND METHODS

The present work is a descriptive study, and the selection of patients were carried out, based on an episode of EKC, who referred to Imam Khomeini Hospital Ahvaz city, Iran, during November to December 2013.

The informed consent was obtained from each patient. This study with the registration number IR.AJUMS.REC.1394.126 was approved by an Ethic Committee of the Deputy Research and Affairs Ahvaz Jundishapur University of Medical Sciences Ahvaz, Iran.

All the patients had clinical signs and symptomatic of acute keratoconjunctivitis, with the signs of sudden redness, discomfort, pain, tearing, follicular reaction, and tender preauricular lymph node enlargement.

Totally, 88 symptomatic patients were considered for this study. The samples were collected from the lower palpebral conjunctiva using sterile Dacron swab, which was subsequently introduced in a transport media (DMEM) supplemented with antibiotics and antifungal (15). Specimens were stored at −70°C for further processing.

### DNA extraction.

High pure viral nucleic acid kit (Roche, Germany) was used for the extraction of DNA according to the manufacturer's instructions.

### Polymerase chain reaction.

PCR is the “gold standard” for diagnosing viral conjunctivitis. Studies indicated that PCR is more sensitive for detecting adenoviruses than other virological methods such as viral culture, antigen detection and serological tests (5, 16).

To perform the test, first GAPDH primers were used as an internal control and to evaluate the quality of extracted DNA. The conserved region within the hexon gene were used in the PCR assay. PCR was performed in 25 μl PCR reaction mixture, containing 7 μl of extracted DNA, 2.5 μl PCR buffer 10×, 0.75 μl MgCl, 0.5 μl dNTP 10 mM, 1U of *Taq* DNA Polymerase, 0.5 μM of each primers of forward, hex1deg (5′-GCC SCA RTG GKC WTA CAT GCA CAT C-3′) and revers, hex2deg (5′-CAG CAC SCC ICG RAT GTC AAA-3′) (17).

The PCR reaction mixture was subjected to thermocycler (Techne TC-5000, UK) and programmed with following condition one cycle: 94°C for 5 min: and 35 cycles: 94°C for 1 min; 56°C for 1 min; 72°C for 1 min, final extension 72°C for 10 min.

### Gel electrophoresis.

The final PCR products were subjected to electrophoresis in 2% agarose gel, stained with safe stain and visualized under ultraviolet transilluminator. The expected size of the PCR products for the HAdV was 301 bp (Fig. 2). DEPC water was used as a negative control and adenovirus 40 and 41 used as a positive control.

### Sequencing.

To confirm the positive samples and determine the HAdV serotypes, randomly six positive samples for partial hexan were sequenced. The sequences were blast using available databases. A phylogenic tree was constructed with Neighbor joining method using the partial nucleotide sequences for partial hexon of detected HAdV. Reference sequences were retrieved from GenBank using their accession numbers.

### Statistical analysis.

Data were statistically defined in terms of median and range or mean and standard deviation (± SD), frequencies. For comparing categorical data, Chi square test was done. A *p* value < 0.05 was considered statistically significant. All statistical calculations were done using SPSS version 21 (SPSS Inc., Chicago, IL, USA).

## RESULTS

**Fig 1.**
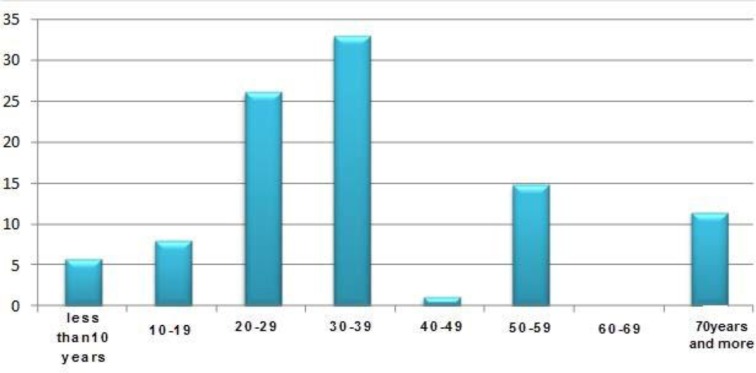
Distribution of age-group patients with EKC

The study population consisted of 39 (44.3%) females and 49 (55.7%) males. The age of the youngest and the oldest patients were 1 and 81, respectively, with the mean average age of 36.15±18.64 (Fig. 1). From the total of 88 specimens tested, 47 (53.4%) were positive in PCR test. Among them 25 (51.02%) were males and 22 (56.41%) females showed positive for HAdV and 41 (46.59%) individuals including 24 (58.5%) males and 17 (41.5%) females were negative for HAdV (P =0.615). Table 1 shows the distribution of adenovirus infection among the age groups of patients. As demonstrated in Table 1, the age group of 30–39 years shows the highest prevalence (17.05%) adenoviral keratoconjunctivitis and the lowest were 2 positive cases in age group of <10 years. However, there was no statistically significant association between the age and adenoviral keratoconjunctivitis (*P*=0.732).

**Table 1.** Distribution of positive and negative HAdV among the age group patients.

**Age group**	**Positive**		**Negative**		**P value**

	**No**	**%**	**No**	**%**	
< 10	3	3.4%	2	2.27%^*^	
10–19	3	3.4%	4	4.54%	
20–29	12	13.63%	11	12.5%	
30–39	15	(17.04%)	14	15.9%^*^	0.732
40–49	0	0	1	1.13%	
50–59	9	10.22%	4	4.54%	
60–69	0	0	0	0	
>70	5	5.68%	5	5.68%	
Total	47	53.4%	41	46.59%	

**Fig. 2.**
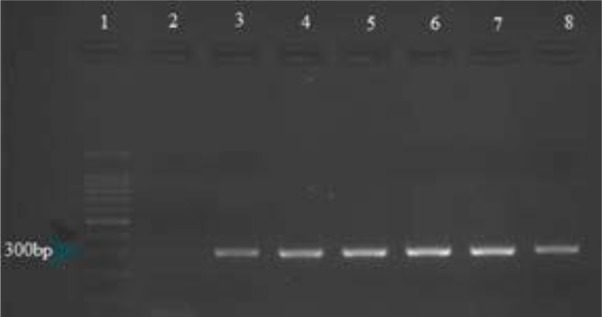
Analysis of the HAdVs Lane 1: 100-bp size marker Lane 2: Negative control Lane 3: Positive control Lane 4–8: samples

The results of sequencing for 6 positive samples revealed that HAdV-8 was the only dominant serotype.

The obtained sequences were blasted using National Center for Biotechnology Information (NCBI) database. The results of blasting showed that all 6 isolated strains had nucleotide identity score of 98% with the standard references of Human Adenovirus D8 retrieved from GenBank.

### Results of Phylogenetic tree.

**Fig. 3.**
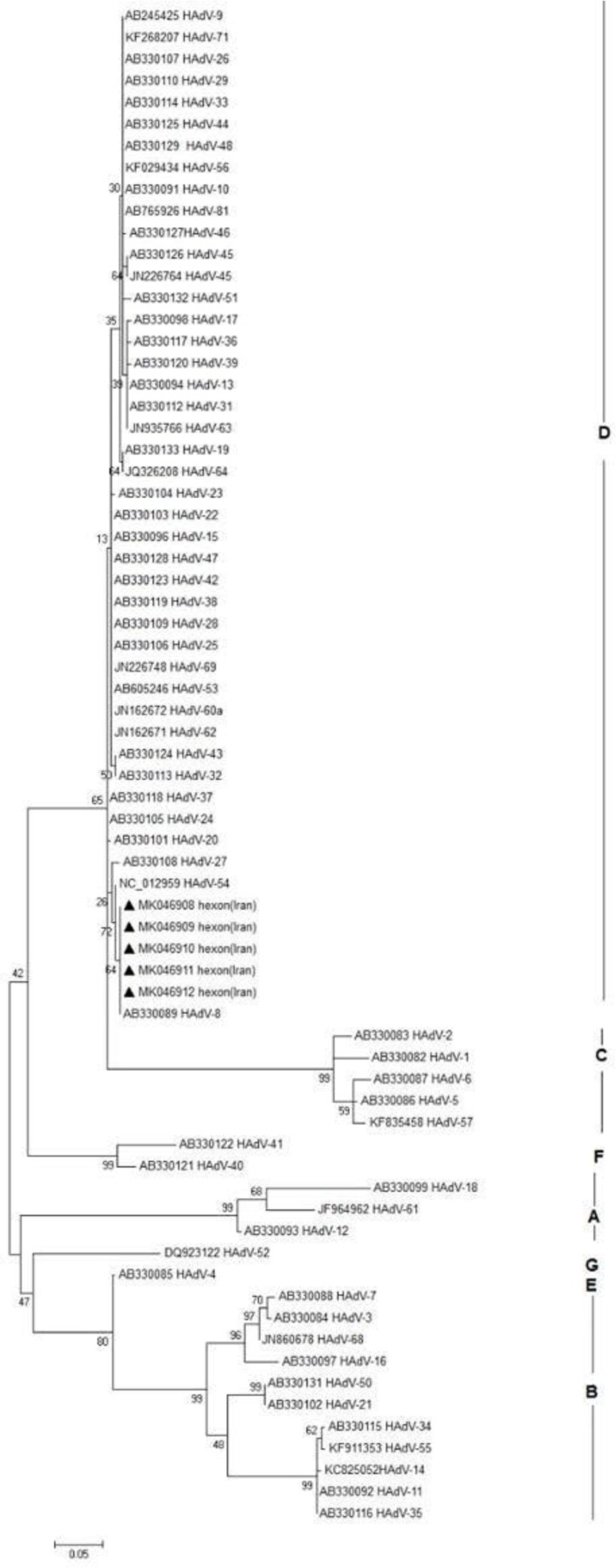
Maximum likelihood tree of the hexon region of e adenovirus serotypes 8 detected in EKC patients with accession numbers MK046908, MK046909, MK046910, MK046911 MK046912 shows in black triangle isolated from Ahvaz, city-Iran. Numbers in branch are reproducibility after 1000 bootstraps. The reference sequences retrieved from GenBank. Scale bar = 0.05

The results of Maximum likelihood tree revealed that the sequences of the all the 6 isolated HAdV8 from patients with acute EKC had very closed nucleotide homology with those isolated HAdV serotype 8 in the different regions of the world. The standard references with their accession number was retrieved from GenBank (Fig. 3).

## DISCUSSION

Epidemic viral conjunctivitis is a very contagious disease that is run into year-round. Adenoviruses and enteroviruses are the main causative agents of EKC (18). Adenovirus represents 15% to 70% of all cases of infectious conjunctivitis in different regions of the world (19). It is the commonest cause of viral conjunctivitis and EKC in ophthalmic clinics in several societies, especially in East and Southeast Asia. Subgroup D adenoviruses including type 8, 19, 37 and adenovirus type 11 (Subgroup B) are the predominant viruses isolated in each endemic outbreak in a different region of the world (20–23).

Three new human adenovirus types that cause EKC were recently detected and named HAdV-53, HAdV-54, and HAdV-56 (20, 22–26). HAdV-56 is a new recombinant type isolated from epidemic keratoconjunctivitis (EKC) patients in the city of Dalian, China (22). In the present study, it was demonstrated that outbreak of EKC was caused by human adenovirus type 8 and it was the only predominant serotype in the patients with EKC in Ahvaz city, Iran. In the current study, 53.4% of the patients with EKC showed positive for HAdV serotype 8 which among them 51.02% were males and 56.41% females (p=0.488).

In blasting process we observed the isolated HAdVs serotype 8 in Ahvaz city had 97% nucleotide identity with HAdV-54, 8 isolated in Japan (23, 25). The Maximum likelihood phylogenetic tree shows the nucleotide homology with the isolated HAdV 8 and the isolates HAdV serotypes 54 in Japan (27).

However, limited data have been reported on the prevalence of EKC in Iran. Sohrabi, et al., conducted a study in Tehran (2014) and described 150 samples of patients with acute conjunctivitis; 22 (14.6%) and 5 (3.3%) cases were considered positive for adenovirus and HSV-1 DNA, respectively (19). Besides, Pinto et al. showed an incidence of 59% for adenovirus in viral conjunctivitis in Brazil which is in agreement with our finding (16). Yagci et al. in Turkey report an incidence of 26.5% for adenoviral conjunctivitis by PCR method (28). The difference probably indicates hygienic and geographical discrepancies between different communities.

Jin et al. conducted a study to investigate the epidemiology of adenoviral conjunctivitis in Hanoi, Vietnam and reported predominantly high prevalence of Ad8 (29). Aoki et al. reported 124/196 (63.26%) of the patients with EKC showed positive for the Ad8 (30). Adenoviral conjunctivitis are largely caused by adenovirus serotypes 3, 4, 8, 11, 19, 22 and 37 (31–33). Ishii et al. compared the clinical and viral data of adenoviral keratoconjunctivitis in Sapporo (Japan), Koahsiung (Taiwan), and Busan (Korea), and found that the most frequently detected agent was Ad 8 (57%), followed by EV 70 (12%), Ad 3 (9%) and Ad 19 (7%). They concluded that Ad8 as the major serotypes in these cities (34).

The results of blasting showed that all the 6 isolated strains had nucleotide identity score of 98% with the standard references of human adenovirus D8 retrieved from GenBank. The Maximum likelihood phylogenetic tree of the nucleotide sequences of the all the 6 isolated HAdV8 from patients with acute EKC showed high degree of Homology with those isolated HAdV serotypes 8 from different regions of the world.

HAdVs that cause EKC are spread person-to-person or by fomites; no vaccines or effective antiviral treatments are available and are resistant to desiccation and certain common surface disinfectants (35, 36).

This study points up the outbreak of HAdV in Ahvaz city, however, effective control measures should be carried out by the health care settings to prevent transmission within clinical settings and the community. Eye care providers should control protocols about EKC, and other infection risks, as recommended by CDC (35). The application of toolkit, patient education, wiping instruments with alcohol pads after each patient contact, daily cleaning of commonly touched surfaces by effective agents against HAdV contamination and a health advisory to providers will reduce HAdV transmission (36).

In conclusion, this study described high prevalence of HAdV among the patients with conjunctivitis, considering that AdV8 was the predominant serotype circulating in Ahvaz city Also, this study is the first report of HAdV outbreak in Iran. Although the role of other viruses such as enteroviruses particularly Coxsakie A24 and Enterovirus 70 are required to be investigated in patients with EKC (37, 38).
